# Botulinum Toxin and Migraine: Goals and Perspectives

**DOI:** 10.3390/toxins16120530

**Published:** 2024-12-10

**Authors:** Maria Pia Prudenzano

**Affiliations:** Headache Center, Neurological Clinic, “L. Amaducci”, AOUC Policlinico, Piazza G. Cesare, 11, 70124 Bari, Italy; mariapia.prudenzano@uniba.it

## 1. Special Issue Introduction

This Special Issue aims to provide an updated overview of the current state and future perspectives of botulinum toxin treatment for migraine. It has been over 14 years since 15 October 2010, when the U.S. Food and Drug Administration approved onabotulinumtoxinA (OBTA) for the prevention of headaches in adults with chronic migraine. Although the toxin has proven to be an effective and well-tolerated therapeutic option, with high patient adherence, several aspects of its use still require in-depth study. In promoting this Special Issue, we asked ourselves the following questions: What is its precise peripheral anti-migraine mechanism of action, and how might it impact the central nervous system? Additionally, what is the optimal injection paradigm? Can long-term treatment modify the natural course of migraine? Could this therapy benefit episodic migraine or other types of headaches? Other interesting aspects to consider included the possibility of shorter administration intervals to counteract dose wear-off and the potential use of botulinum toxin alongside the anti-Calcitonin Gene-Related Peptide (anti-CGRP) therapy in resistant cases. The Calcitonin Gene-Related Peptide (CGRP) is a vasoactive neuropeptide which plays a well-recognized role in the pathogenesis of migraine, providing a strong rationale for the development of drugs targeting it [1,2]. The meningeal vasculature receives a dense innervation of both unmyelinated C-fibers and thinly myelinated Aδ fibers nociceptive afferent fibers which stem from the trigeminal nerve [2]. The neuropeptide CGRP is highly expressed in trigeminal ganglion neurons and is released from peripheral and central nerve terminals, as well as being secreted within the trigeminal ganglion itself [2]. The release of the CGRP from peripheral terminals triggers a series of events, including increased nitric oxide production and sensitization of trigeminal nerves [2]. Within the trigeminal ganglion, the secreted CGRP interacts with nearby neurons and satellite glial cells, sustaining peripheral sensitization and potentially driving central sensitization in second-order neurons [2]. When OBTA is introduced into the extracellular space, its heavy chain binds to receptors on the C-fiber nerve terminal. The toxin is then internalized via endocytosis, becoming enclosed in a vesicle within the nerve terminal [3]. Once inside, the light chain separates from the heavy chain and moves into the cytoplasm, where it cleaves Synaptosomal-Associated Protein-25 (SNAP-25), a key protein involved in the fusion of vesicles containing neuropeptides with the nerve terminal membrane [3]. This prevents the release of the CGRP and other neuropeptides [3]. Additionally, the insertion of certain receptors, such as Transient Receptor Potential Vanilloid-1 (TRPV1) and Purinergic Receptors 2X3 (PR2X3), which are transported to the cell membrane via vesicles, is also inhibited [3]. OBTA has been shown to selectively inhibit unmyelinated C-fibers, without affecting Aδ-meningeal nociceptors [4]. In contrast, anti-CGRP monoclonal antibodies (mAbs) that target the CGRP have been demonstrated to block the activation of Aδ-fibers but not C-fibers [5]. Therefore, the combined use of anti-CGRP mAbs and onabotulinumtoxinA (onabot) may provide a synergistic effect by targeting both Aδ-fibers and C-fibers. The contributions published here provide answers to many of these questions and offer insights for further research.

## 2. An Overview of Published Articles

The retrospective observational study by Giulia Ceccardi et al. (Contribution 1) shows that chronic migraine patients treated preventively with botulinum toxin, compared to patients previously treated with traditional drugs, exhibit a better response to anti-CGRP therapy after three months, as measured by reduced migraine days and Migraine Disability Assessment Score (MIDAS) ratings. Furthermore, the response to monoclonal antibodies is more evident in patients pre-treated with at least three cycles of botulinum toxin compared to those with shorter treatment durations. Although the study’s retrospective design, small sample size, and brief observation period represent limitations, the authors highlight three key findings: First, botulinum toxin appears to revert migraines from chronic to episodic, reducing allodynia—a result not seen in patients pre-treated with oral preventive therapies. Second, botulinum toxin’s inhibition of CGRP release may facilitate the action of anti-CGRP treatments, especially those targeting the ligand. Lastly, OBTA and anti-CGRP antibodies seem to have a synergistic effect, supporting the combination of both treatments in selected cases, as suggested by previous studies.

The prospective observational study by Angelo Torrente et al. (Contribution 2) focuses on the importance of sleep quality for overall well-being, noting that this parameter should always be included in migraine treatment outcomes, even for patients without overt sleep disturbances. Starting from the premise that sleep and migraines influence each other bidirectionally, the authors propose that improving one should improve the other, and vice versa. Torrente et al. demonstrate, that while a single session of botulinum toxin therapy does improve migraines in over 60% of patients, it does not significantly reduce Pittsburgh Sleep Quality Index (PSQI) scores or anxiety and depression scores correlated with the PSQI. This result suggests that the relationship between sleep, emotional state, and migraine is more complex than a simple indirect correlation and that significant improvement in sleep quality requires long-term migraine stabilization and the absence of negative emotional influences.

One of the most intriguing goals of clinical research is the identification of potential predictive factors for treatment response. Daniele Martinelli et al.’s study (Contribution 3) uses a Machine Learning (ML) algorithm to retrospectively analyze the anamnesis characteristics of excellent responders and non-responders to botulinum toxin. None of the parameters analyzed could distinguish between responders and non-responders among patients with chronic migraine. However, in patients with high-frequency episodic migraine (HFEM), the age of migraine onset and the Hospital Anxiety and Depression Scale (H. A. D. S.) subscore for anxiety positively correlated with the response to botulinum toxin, while opioid use negatively correlated with treatment response. Disability level, measured by the MIDAS scale, also correlated negatively with response but did not reach statistical significance. These results, particularly in the HFEM group, are clinically important as they provide guidance on botulinum toxin therapy for patients at risk of chronic migraine progression.

Francesco Bono et al. (Contribution 4) conducted a randomized, double-blind, placebo-controlled study demonstrating the efficacy and safety of the “follow the origin of maximum pain” approach using subcutaneous OBTA injections in the occipital or trigeminal skin area in chronic migraine patients who did not respond to intramuscular OBTA injections. Both groups treated in the occipital and trigeminal skin areas showed a significant reduction in headache days, pain intensity, and disability compared to the placebo. The authors emphasize that the maximum pain origin area also identifies the site of allodynia, an expression of sensitization and that the toxin has previously shown to inhibit both peripheral and indirectly central sensitization.

The real-world study by Claudia Altamura et al. (Contribution 5) evaluates a broader range of outcome measures than just migraine frequency changes, including symptomatic medication use, pain intensity, and migraine-related disability. The study highlights therapeutic responses from the first treatment cycle, with progressive improvement over a year. Even patients who do not achieve a 50% or 30% reduction in migraine days report significant reductions in attack intensity, describing it as non-disabling. The authors note that this parameter is often overlooked when considering preventive migraine therapy outcomes. Another noteworthy finding is that patients treated with OBTA experience rapid reductions in throbbing pain, which is associated with high disability scores, aligning with the hypothesis that OBTA acts on C fibers releasing CGRP that are responsible for slow-building pain like aching, throbbing, or burning.

Licia Grazzi et al. (Contribution 6) explore pain catastrophizing, which is often overlooked in chronic migraine therapy evaluation. Defined as a range of negative emotional responses to anticipated or actual pain, pain catastrophizing includes persistent intrusive thoughts, exaggerated worry, and feelings of helplessness. This longitudinal open-label study on chronic migraine patients with Medication Overuse Headaches shows improved headache frequency and medication intake after six months of treatment, with benefits maintained at 12 months. Moreover, pain catastrophizing, particularly helplessness, predicts decreased headache frequency and medication use over 12 months. The authors hypothesize that therapy helps patients manage acute treatments more effectively, limiting the number of headache days.

Gabriele Sebastianelli et al. (Contribution 7) study the neurophysiological correlates of botulinum toxin therapy through recordings of the nociceptive blink reflex (nBR), the trigeminocervical reflex (nTCR), pain-related cortical evoked potential (PREP), and upper limb somatosensory evoked potential (SSEP). Results show that a single OBTA session significantly reduces headache days, pain intensity, and analgesic use. Electrophysiological test changes indicate that OBTA exerts a neuromodulatory effect on the trigeminal system by reducing input from meningeal and other trigeminovascular nociceptors, as well as decreasing cortical pain-processing activity to restore normal descending pain modulation.

The multicenter prospective observational “real-life” study by Ilenia Corbelli et al. (Contribution 8) demonstrates the long-term efficacy and safety of onaBT-A treatment in chronic migraine (CM) patients over two years, with a high adherence rate. Among patients, 52.3% were responders, 17.9% were poor responders, 15.4% were non-responders, and 14.4% discontinued. The treatment was generally well-tolerated, with most adverse events consisting of a sensation of contraction with cervicobrachialgia, diffuse muscle pain, or localized pain at the injection site. Only three serious adverse events were reported—one pregnancy, one death, and one hospitalization—none of which were related to OBTA treatment. A potential study bias is the fact that 48.2% of enrolled patients were also using concurrent oral prophylaxis. Although no new oral preventive treatment was introduced, modifications (dose adjustments) of ongoing therapies were allowed in the second year of treatment. The high adherence rate indirectly supports the treatment’s efficacy and tolerability, while also reflecting patient preferences for this administration route.

Dilara Onan et al.’s retrospective open-label real-world study (Contribution 9) uniquely investigates neck pain, which is an important pain component in CM that may arise from trigemino-cervical sensitization. The authors analyzed the 3-month effects of a single OBTA treatment session on neck pain and headaches in CM patients. Results showed that a single session reduced neck pain, headache intensity, monthly headache days, and disability in daily life while improving quality of life over 3 months.

Angelo Torrente et al. (Contribution 10) explored multisensory perception using the sound-induced flash illusion, finding that OnabotulinumtoxinA effectively prevents migraines and reduces headache severity. Even a single therapy session could partially restore multisensory processing, as indicated by patients’ susceptibility to the illusion. OBTA may modulate cortical excitability in migraine patients alongside its positive effects on headache prevention.

The narrative review by Carlo Baraldi et al. (Contribution 11) addresses the pathophysiology of migraine and examines current knowledge on OBTA ‘s mechanism of action and efficacy in migraine management. The authors emphasize that OBTA is one of the few treatments with a specific indication for CM, as well as the first treatment approved for this headache type. Reviewing studies up to 30 September 2022, they highlight evidence that OBTA reduces monthly headache days, enhances quality of life, and is associated with lower health service use, particularly in emergency settings. While OBTA ‘s true anti-nociceptive mechanism in CM remains uncertain, its central effects are a point of investigation.

## 3. Conclusions

The studies in this Special Issue confirm the efficacy and tolerability of Onabotulinum Toxin Type A (OBTA) in chronic migraine therapy. OBTA improves traditional preventive treatment outcomes, including a reduction in headache days and intensity, fewer symptomatic medication days, and enhanced migraine-related disability and quality of life. The findings from these contributions are substantiated by recent evidence [6,7]. A systematic review and network meta-analysis highlights that, among all preventive treatments for chronic migraine, Botulinum toxin A stands out with the most favorable efficacy and safety profile, followed closely by monoclonal antibodies targeting the calcitonin gene-related peptide (CGRP) [6]. A pooled analysis of the Phase 3 REsearch Evaluating Migraine Prophylaxis Therapy (PREEMPT) randomized controlled studies shows that the proportions of participants with chronic migraine achieving <15 monthly headache days with onabotulinumtoxinA were 60.9% (419/688) at weeks 25–56, 81.1% (558/688) at weeks 53–56, and 79.4% (546/688) during any consecutive 12-week period [7]. Patients who showed a reduction in their monthly headache days to fewer than 15 experienced significant improvements in both headache-related disability and migraine-specific quality of life, compared to those whose headache days remained at or above the 15-day threshold. The sustained benefits observed over a 56-week period underscore the long-term effectiveness of onabotulinumtoxinA in preventing chronic migraine [7].

Other treatment outcomes, such as neck pain—a frequent CM component that may lead to misdiagnosis—pain catastrophizing, helplessness, sleep quality, and patients’ perceived improvement, are also examined. Notably, many outcomes show improvement from the first administration, with some measures, such as sleep quality and negative emotional states, requiring longer treatment periods. A systematic review and meta-analysis reveals a higher level of neck pain-related disability in migraine patients compared to those with tension-type headache (TTH), highlighting the particular relevance of botulinum toxin’s effectiveness on this pain component [8]

Two studies provide insights into the neurophysiological correlates of the clinical improvements induced by OBTA through a neuromodulatory effect on the trigeminal system, potentially reducing inputs from meningeal and other trigeminovascular nociceptors and influencing cortical excitability. Other evidence suggesting a primary central modulatory action of OBTA comes from a pilot prospective cohort study using high-density electroencephalography (HD-EEG) [9]. The study shows that chronic migraine patients are characterized by a disrupted EEG-Functional Connectivity compared to controls and that a single session of OBTA treatment is able to restore it.

A new paradigm for subcutaneous administration is proposed, which may benefit non-responders to the classic injection paradigm. This new injection paradigm, like other paradigms proposed previously [10], should be evaluated in studies that can compare it with the traditional PREEMPT approach.

Efforts to identify a predictive clinical parameter for treatment response through a machine learning (ML) algorithm yielded inconclusive results for CM, but laid groundwork for extending OBTA’s indication to high-frequency episodic migraine patients at risk of chronicization.

Several contributions in this Special Issue also provide results and research avenues for combining OBTA with anti-CGRP drugs, suggesting that pretreatment with BT-A, even when not fully effective, may positively impact subsequent anti-CGRP therapy, potentially by reducing allodynic components [11,12,13,14,15].

These findings encourage further research to confirm these results, offer new perspectives on OBTA use in migraines and other headache types, and deepen the understanding of its mechanism of action and central influences.

## References

[1] Dodick D.W. (2018). A Phase-by-Phase Review of Migraine Pathophysiology. Headache.

[2] Iyengar S., Johnson K.W., Ossipov M.H., Aurora S.K. (2019). CGRP and the Trigeminal System in Migraine. Headache.

[3] Becker W.J. (2020). Botulinum Toxin in the Treatment of Headache. Toxins.

[4] Zhang X., Strassman A.M., Novack V., Brin M.F., Burstein R. (2016). Extracranial injections of botulinum neurotoxin type A inhibit intracranial meningeal nociceptors’ responses to stimulation of TRPV1 and TRPA1 channels: Are we getting closer to solving this puzzle?. Cephalalgia.

[5] Cohen F., Yuan H., De Poy E.M.G., Silberstein S.D. (2022). The Arrival of Anti-CGRP Monoclonal Antibodies in Migraine. Neurotherapeutics.

[6] Zhao C., Li C., Yu X., Dai X., Zou W. (2024). Effectiveness and safety of pharmacological prophylaxis for chronic migraine: A systematic review and network meta-analysis. J. Neurol..

[7] Silberstein S.D., Diener H.C., Dodick D.W., Sommer K., Lipton R.B. (2024). Sustained benefits of onabotulinumtoxinA treatment in chronic migraine: An analysis of the pooled Phase 3 REsearch Evaluating Migraine Prophylaxis Therapy (PREEMPT) randomized controlled trials. Headache.

[8] Al-Khazali H.M., Al-Sayegh Z., Younis S., Christensen R.H., Ashina M., Schytz H.W., Ashina S. (2024). Systematic review and meta-analysis of Neck Disability Index and Numeric Pain Rating Scale in patients with migraine and tension-type headache. Cephalalgia.

[9] Conti M., Bovenzi R., Palmieri M.G., Placidi F., Stefani A., Mercuri N.B., Albanese M. (2024). Early effect of onabotulinumtoxinA on EEG-based functional connectivity in patients with chronic migraine: A pilot study. Headache.

[10] Stovner L.J., Hagen K., Tronvik E., Bruvik Gravdahl G., Burstein R., Dodick D.W. (2022). FollowTheSutures: Piloting a new way to administer onabotulinumtoxinA for chronic migraine. Cephalalgia.

[11] Pellesi L., Do T.P., Ashina H., Ashina M., Burstein R. (2020). Dual Therapy with Anti-CGRP Monoclonal Antibodies and Botulinum Toxin for Migraine Prevention: Is There a Rationale?. Headache.

[12] Blumenfeld A.M., Frishberg B.M., Schim J.D., Iannone A., Schneider G., Yedigarova L., Manack Adams A. (2021). Real-World Evidence for Control of Chronic Migraine Patients Receiving CGRP Monoclonal Antibody Therapy Added to OnabotulinumtoxinA: A Retrospective Chart Review. Pain Ther..

[13] Scuteri D., Tonin P., Nicotera P., Vulnera M., Altieri G.C., Tarsitano A., Bagetta G., Corasaniti M.T. (2022). Pooled Analysis of Real-World Evidence Supports Anti-CGRP mAbs and OnabotulinumtoxinA Combined Trial in Chronic Migraine. Toxins.

[14] Guerzoni S., Baraldi C., Pani L. (2022). The association between onabotulinumtoxinA and anti-CGRP monoclonal antibodies: A reliable option for the optimal treatment of chronic migraine. Neurol. Sci..

[15] Salim A., Hennessy E., Sonneborn C., Hogue O., Biswas S., Mays M., Suneja A., Ahmed Z., Mata I.F. (2024). Synergism of Anti-CGRP Monoclonal Antibodies and OnabotulinumtoxinA in the Treatment of Chronic Migraine: A Real-World Retrospective Chart Review. CNS Drugs.

